# Egyptian metallic inks on textiles from the 15^th^ century BCE unravelled by non-invasive techniques and chemometric analysis

**DOI:** 10.1038/s41598-019-43655-z

**Published:** 2019-05-13

**Authors:** G. Festa, T. Christiansen, V. Turina, M. Borla, J. Kelleher, L. Arcidiacono, L. Cartechini, R. C. Ponterio, C. Scatigno, R. Senesi, C. Andreani

**Affiliations:** 1 CENTRO FERMI - Museo Storico della Fisica e Centro Studi e Ricerche “Enrico Fermi”, Piazza del Viminale 1, 00184 Rome, Italy; 2 CNR – Istituto per i Processi Chimico-Fisici (IPCF), Viale Ferdinando Stagno d’Alcontres 37, Messina, Italy; 3 CNR- Istituto di Scienze e Tecnologie Molecolari (ISTM), Via Elce di sotto 8, 06123 Perugia, Italy; 4 Museo Egizio di Torino, Via Accademia delle Scienze 6, 10123 Turin, Italy; 5 Supreme Council for Archeology of Piedmont, P.zza S. Giovanni 2, 10122 Torino, Italy; 6 STFC, Rutherford Appleton Laboratory - ISIS neutron and muon Facility, Didcot, OX11 0QX United Kingdom; 7 University College London, Institute of Archaeology, 31-34 Gordon Square, Kings Cross, London, WC1H 0PY United Kingdom; 8 Università degli Studi di Roma Tor Vergata, Dipartimento di Fisica and NAST Centre, Via della Ricerca Scientifica 1, 00133 Rome, Italy; 9 Università degli Studi di Roma Tor Vergata, Dipartimento di Scienze e Tecnologie Chimiche, Via della Ricerca Scientifica 1, 00133 Rome, Italy

**Keywords:** Anthropology, Characterization and analytical techniques

## Abstract

The development of black inks has enabled writing to become an established method of communication in history. Although a large research effort has been devoted to the study of pigments and dyes used in ancient Egypt to decorate burial walls and furnishings, or to write on papyrus, to date little attention has been paid to the nature and technology of inks used on ritual and daily-use textiles, which may have fostered the transfer of metallic ink technology onto papyrus and parchment supports. We report about inks from 15^th^ century BCE Egyptian textiles by combining non-invasive techniques, including ultraviolet (UV) reflected imaging, near-infrared reflectography (NIRR), X-ray fluorescence (XRF) spectroscopy, Raman spectroscopy and prompt-gamma-activation-analysis (PGAA). It is argued that the inks are related to the family of iron gall inks, whose introduction is commonly attributed to the third century BCE. This interpretation frames the technology of writing on fabrics, used by the ancient Egyptians, in a different time, thus providing new information on the genesis of mordant inks in the ancient Mediterranean cultures. We anticipate our study to be a starting point for further and more sophisticated investigations of textiles, which will clarify the origin of metallic ink in the ancient world.

## Introduction

Black ink is the established and time-honoured way in which humankind commits words to writing and communicates thoughts and experiences over short and vast distances of time and space. There can be no doubt that this ground-breaking invention can be traced back to the beginning of “history” in ancient Egypt (c. 3200 BCE). Only in recent years has this invention and its history in ancient Egypt and the related Mediterranean cultures been the subject of systematic scientific studies and analyses^1–5^. To date the focus has been on black ink inscribed on the papyrus medium, while other writing surfaces like for instance textile have been neglected.

Here, we report on the chemical composition of black ink inscribed on ancient Egyptian linen using non-invasive methods: Ultraviolet (UV) reflected imaging, near infrared reflectography (NIRR), X-ray fluorescence (XRF) spectroscopy, Raman spectroscopy and prompt gamma activation analysis (PGAA). In total, the research is conducted on a corpus of 19 inscribed textiles. 18 of the textiles derive from the tomb of a royal architect named Kha. He lived during the reigns of the pharaohs, Amenhotep II, Thutmose IV and Amenhotep III (c. 1425–1353 BCE). The tomb of Kha and his wife, Merit, was discovered in 1906 in the necropolis of Deir el-Medina – located near Luxor – by the Italian Egyptologist Ernesto Schiaparelli (1856–1928). Besides 150 linen textiles, the grave goods, which today are primarily housed at the Museo Egizio in Turin, included wooden coffins and furniture, alabaster and metallic vessels, pottery and papyrus and so on^6^. The textiles in question are inscribed either with the name of Kha or his emblematic sign (“identity mark”^7^) – eight of them are loincloths and ten of them tunics (cf. Table 1–S1). Some of the loincloths are shabby and threadbare, indicating that most of them had been worn by Kha during his lifetime. Texts on papyri and limestone flakes (ostraca) from Deir el-Medina tell us that noblemen had their clothes washed by a designated group of people – an ancient “laundry service” of sorts^6,8,9^. Therefore, the inscriptions at the edge of the cloths served the practical purpose of identifying the rightful owner of the garments. It is evident that for this reason an ink solution that could withstand water and friction was needed.

**Figure 1:**
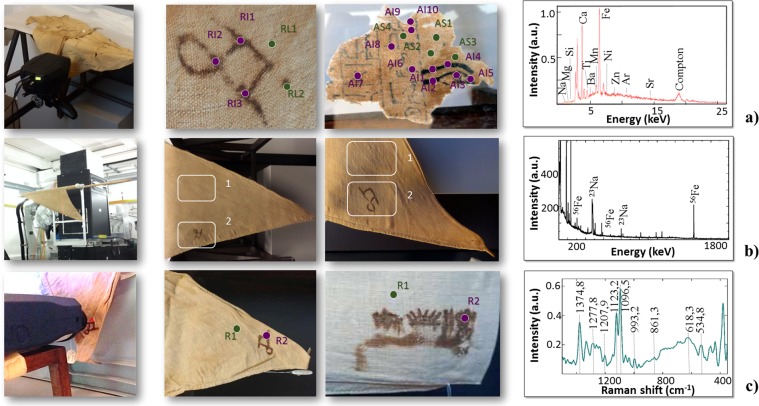
Measurements of the elemental, isotopic and molecular composition of the inks on ancient Egyptian linen textiles using X-ray, neutron and Raman techniques: (**a**) X-ray fluorescence (XRF). From left to right: visible light pictures of the textiles during XRF measurements, XRF measurement points and an XRF spectrum; (**b**) prompt gamma activation analysis (PGAA). From left to right: visible light pictures of the textiles during PGAA measurements, PGAA measurement areas and a PGAA spectrum; (**c**) Raman spectroscopy. From left to right: visible light pictures of the textiles during Raman measurements, Raman measurement areas and a spectrum.

Today, the inks have a brownish appearance and have corroded the linen fibres in most cases. This evidence suggests that the ink’s composition is responsible for the corrosion and the piercing of the textiles. These features are characteristic of iron gall ink, which is primarily made by mixing oak galls (containing gallotannic acid) with an iron sulphate (FeSO_4_•7H_2_O). This mixture produces a writing fluid that on exposure to air turns black through oxidation^10^. The first definite evidence for the use of iron gall ink dates to the late third century BCE, where it is described as a novelty by Philo of Byzantium^11^, whereas results of this study show how the inks on the garments of Kha are related to iron gall ink. Thus, the technology of producing ink based on ferrous gallotanate is much older than expected. The results from the analyses of linen of Kha are compared to an unpublished fragment of an inscribed funerary shroud in the Museo Egizio that dates to c. 1550 BCE (S.5065/2). Unlike the cloths of Kha, this textile was not intended for wear, but was used as a covering for the mummy. The textile is inscribed with extracts from the so-called “Book of the Dead” – a collection of spells which allowed the deceased to navigate through the regions of the netherworld^12^. This fragment was studied using UV/NIRR imaging and XRF spectroscopy. Previous analyses carried out at the British Museum of the black inks used on a comparable shroud also from c. 1550 BCE, revealed the presence of two types of black ink. One was based entirely on amorphous carbon and the other on a mixture of amorphous carbon and a manganese compound, likely manganite (MnO[OH])^13^. Three experimental campaigns are carried out using UV reflected imaging, NIRR, portable XRF and Raman instruments (*in-situ* measurements) at the Museo Egizio in Turin. These are complemented with neutron measurements at the Science and Technology Facilities Council (STFC), ISIS Spallation Neutron Source at the IMAT beamline^14–17^. A sketch of the experiments is reported in Fig. 1 below, and a full description of the measurements is reported in the supplementary information (SI). Table S1 reports the whole set of measurement points (MPs) for the XRF spot analysis, which is divided into three main classes (MP on unwritten tunics, MP on unwritten loincloths and MP on inscriptions), and a description of the assigned labels identifying the MPs for each of the investigated objects.

## Results

### Ink characterisation using light and neutron probes

**Figure 2:**
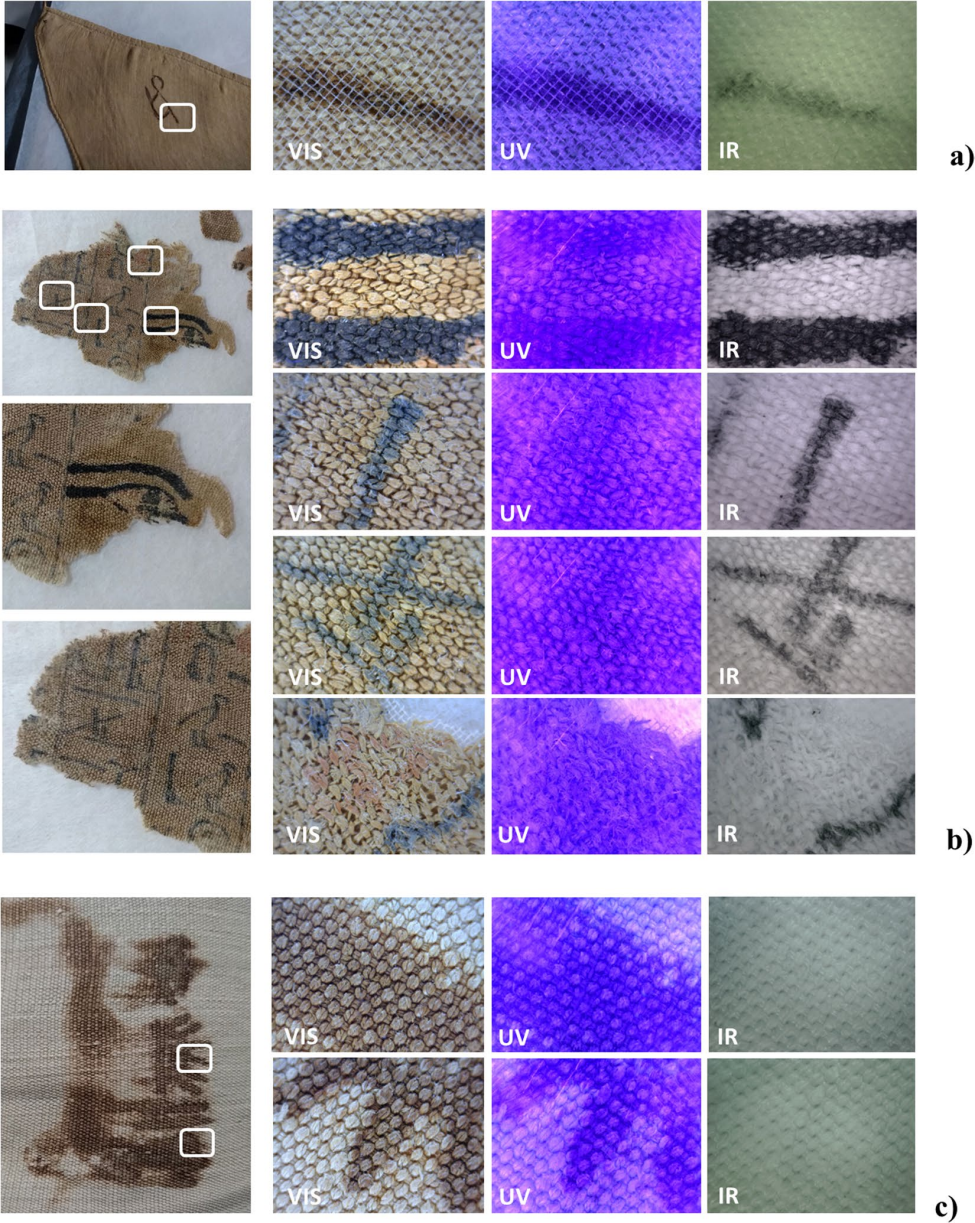
Measurements of the UV/NIR imaging of the inks on ancient Egyptian linen textiles. (**a**) S.8578; (**b**) S.5065/2; (**c**) S.8536.

A preliminary characterisation of the inks inscribed on four textiles (S.5065/2, S.8535, S.8536 and S.8578) is done using UV imaging and NIRR. The black ink on the funerary shroud (S.5065/2) appears black at 940 nm and shows no signs of transparency in the NIR region. This suggests that it is carbon-based. XRF imaging analysis was performed on the same textile in order to reveal the 2D elemental distribution of heavy elements, using a movable set-up with a maximum lateral resolution of 35 μm^18^. This showed that the black ink contained manganese (Mn) and small amounts of iron (Fe), so likely it is based on a combination of amorphous carbon and a Mn(Fe) compound like the Norwich shroud^13^. Unlike the funerary shroud (S.5065/2), the textiles from the tomb of Kha turned partly semi-transparent and partly invisible in the NIR region (S.8578, S.8535 and S.8536). The first phenomenon – semi-transparency – could indicate the use of an iron-gall ink, but the second phenomenon – invisibility – seems to exclude this possibility and rather suggests that the inks are based on ochre (see Fig. 2)^19–21^.

In order to investigate the elemental and molecular composition of these unique inks in a statistically significant set of textiles, an extended campaign of measurements is carried out using XRF and Raman spectroscopy on a set of 18 inscribed linen garments from the tomb of Kha. A total of 15 chemical elements have been identified through the XRF measurements: sodium (Na), magnesium (Mg), aluminium (Al), silicon (Si), sulphur (S), potassium (K), calcium (Ca), titanium (Ti), chromium (Cr), Mn, Fe, copper (Cu), zinc (Zn), arsenic (As) and strontium (Sr). The analysis of XRF peaks indexing, carried out on the three classes of MP, shows that:

1. Fe, Mn and K are the main elements of the ink,
2. Ca is evenly distributed in the linen textile.

Raman spectroscopy shows a signal enouncement in the range of 600–800 cm^−1^ (see Fig. 1c and SI) indicating the presence of iron oxides and oxyhydroxides^22^.

Further, a complementary neutron-based characterisation is carried out to measure the isotopic composition of the textiles, at the STFC-ISIS Spallation Neutron and Muon Source, using the IMAT beamline. PGAA is carried out on two textiles (S.8576 and S.8578), aiming at the isotopic/elemental analysis of the inks. In the first textile (S.8576) eight isotopes are detected: ^24^Mg, ^31^P, ^35^Cl, ^39^K, ^56^Fe, ^63^Cu, ^65^Cu, ^75^As. In the second textile (S.8578) six isotopes are detected (S.8578): ^24^Mg, ^31^P, ^56^Fe, ^63^Cu, ^65^Cu, ^75^As (see Table S2).

### Evidence of the metallic nature of the inks

XRF results from the 143 measurements are organised in a matrix, which is subdivided in three submatrices corresponding to the following categories:

- Unmarked linen areas on tunics (37 MPs)
- Unmarked linen areas on loincloths (24 MPs)
- Inscriptions (82 MPs)

**Figure 3:**
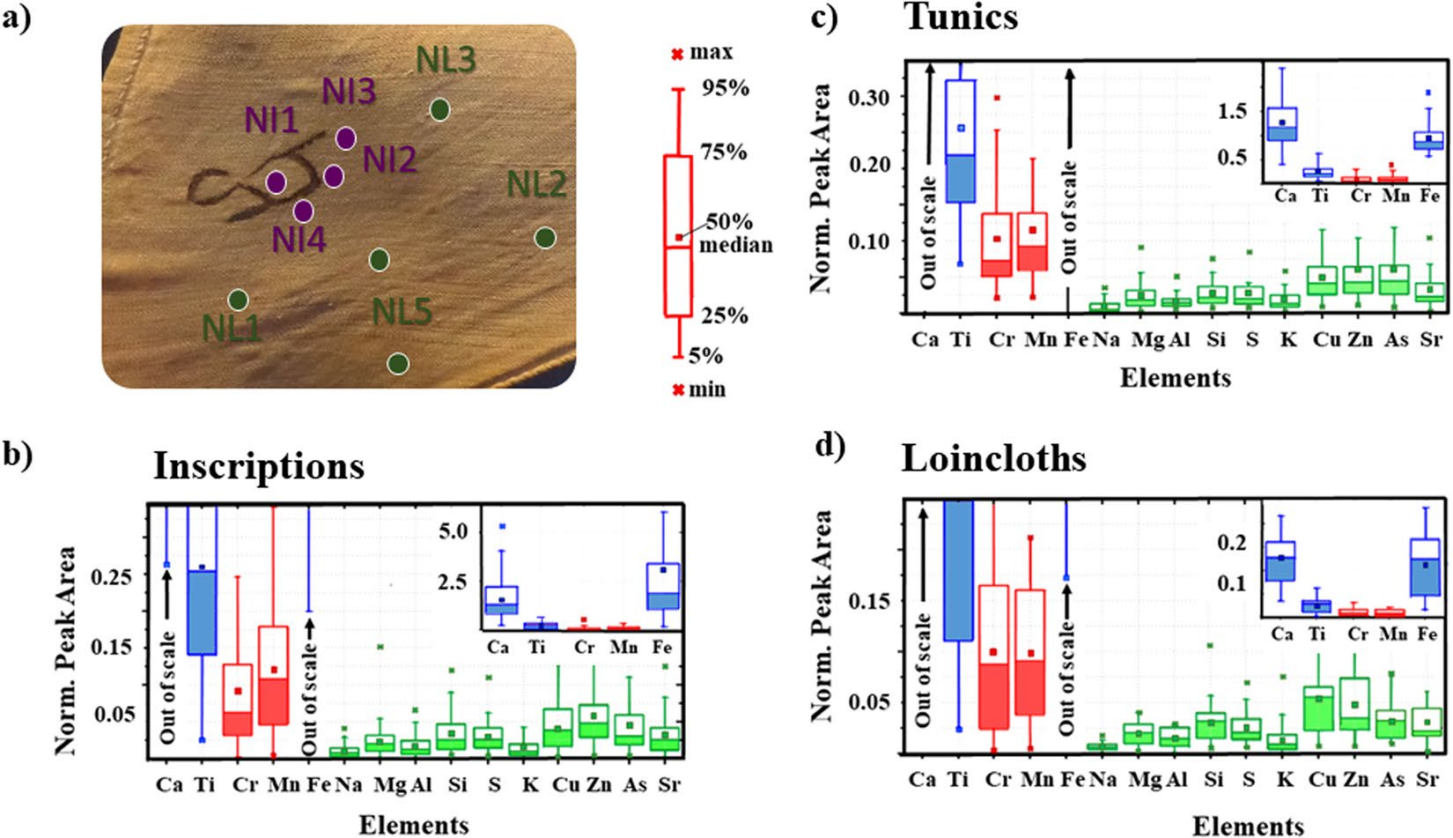
Visible light photograph example with measurement points, and “Box and Whiskers” plots for the chemical elements measured through the XRF analysis of the textiles. (**a**) is a visible light photograph of the linen S.8578 with superimposed four measurement points (NI1-NI4) in the iron-manganese rich inscription areas depicting Kha’s monogram, and five measurement points (NL1-NL5) in the unmarked linen areas on the loincloth (see Supplementary Information for details); (**b**–**d**) show “Box and Whiskers” results for measurement points from unmarked linen areas on tunics (referred to as Tunics), from all inscriptions (referred to as Inscriptions) in tunics and loincloths, and unmarked linen areas on loincloths (referred to as Loincloths), respectively. The box for each element represents the statistical distribution of the element’s XRF peak area: the lower and upper sides of the box indicate lower and upper quartiles, respectively. Inside each box (see the legend sketch in **a**)), the solid horizontal lines indicate median, and squares indicate the mean values. The length of whiskers indicates 5th to 95th percentile range. Outside each box, small crosses indicate minimum and maximum values. Elements detected in the Inscriptions showing the highest peak values (above 0.2) and larger variances are highlighted in blue colour (Ca, Ti and Fe); elements detected in the Inscriptions with intermediate peak intensities (above 0.05 and below 0.2) and variances are highlighted in red colour (Cr, Mn), while elements detected in the Inscriptions with small peak intensities (below 0.05) and small variances are highlighted in green colour (Na, Mg, Al, Si, S, K, Cu, Zn, As and Sr). XRF spectra are normalised by dividing each net count area by the Compton peak area.

The correlation between the presence and abundance of Fe, Mn and K in XRF spectra from a generic MP of typical spot size of 1 mm diameter, and their correspondence to the spatial distribution of the ink, is derived using “Box & Whiskers” representations and principal component analysis (PCA) (details are reported in SI)^23–26^. Box plots, shown in Fig. 3, illustrate the abundance of elements of the three identified categories, respectively. Linen tunics and loincloths present a similar elemental composition or “fingerprint” pattern (Ca, Fe and Ti being the major elements)^27^. In the tunics (Fig. 3a) Fe shows a smaller variance than in the loincloth measurement points (Fig. 3b). In Fig. 3c the elemental variability in the black/brownish inscriptions of the loincloths is shown. In this case, the main element of the category is Fe, and it is characterised by a marked difference between mean and median values of the relative abundance across the distribution of the MPs. The asymmetrical distribution may arise from the non-uniform ink transfer onto the fibres by the nib during writing. A cross correlation analysis is also performed on the detected chemical elements in the inscribed areas of the textiles. The amounts of Fe and Mn show the highest correlation factor (R = 0.86), as compared to the others possible associations among the detected elements (As and S, Fe and S), indicating the presence of a Fe/Mn based ink. A 0.46–0.48 R-factor of correlation between the main elements (Fe, Mn, Cr and Ti) and S is also observed, suggesting a pioneer mixture of a compound with some affinities to iron gall ink.

Principal component analysis (PCA) is carried out on the whole set of XRF data to determine the correlations among the detected chemical elements and spot outliers, using the following variables:

(i) The list of measurement points and division into the inscription category
(ii) The different detected elements associated with the XRF peaks
(iii) The peaks’ intensities (integrated counts)^24–26^

**Figure 4:**
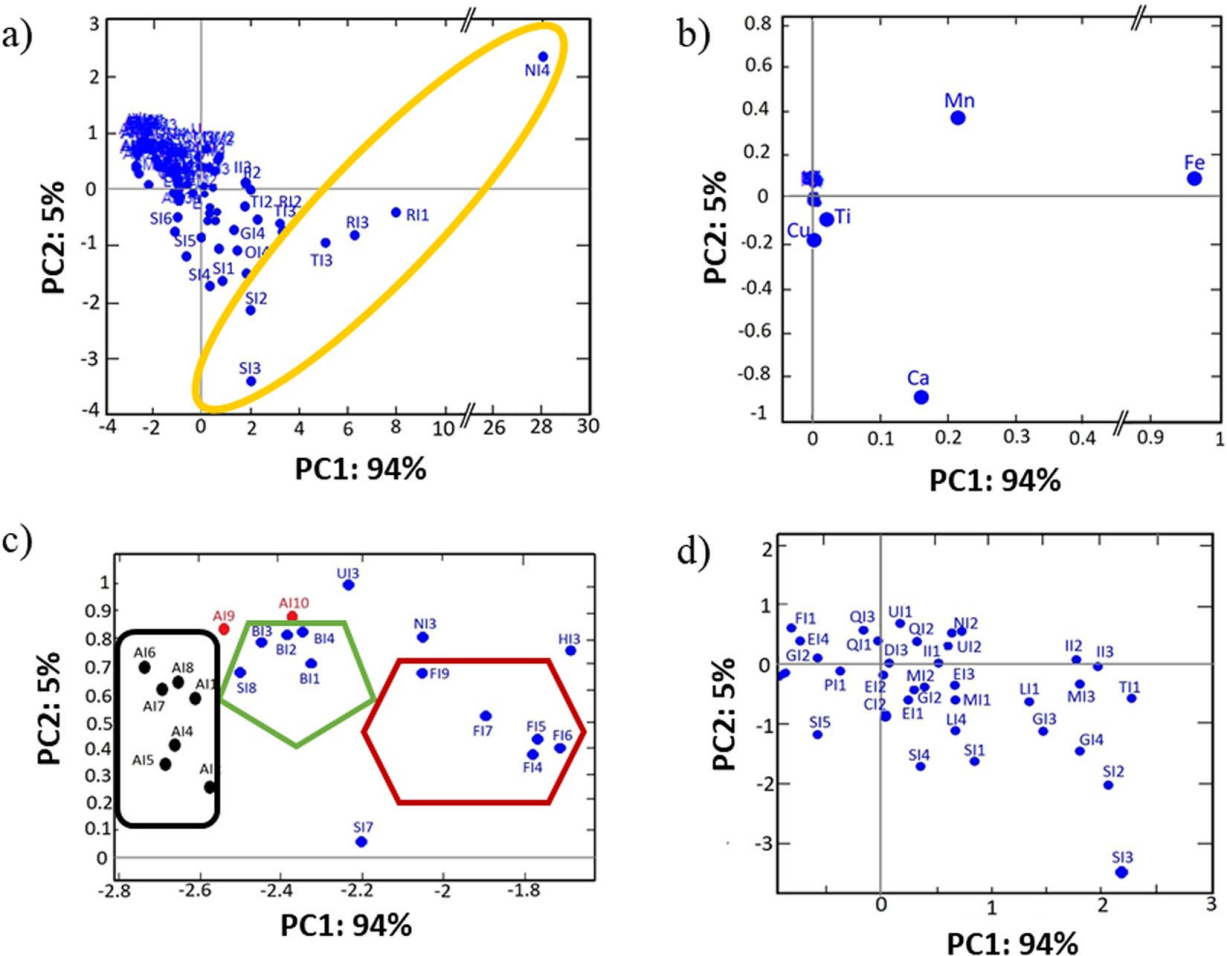
Principal component analysis (PCA) of the XRF measurements on the inscriptions. (**a**) PCA loading plot of the elemental composition (see SI for the list of MPs and their assignments). The yellow ellipse highlights the six points with uniform composition with the largest quantity of detected ink. (**b**) PCA scores plot of the elements; Fe and Mn are well separated from the rest confirming their high correlation values (R = 0.86). The PC1 in (**a**,**b**) is attributed to the amount of ink released during writing. (**c**) Enlargement of the upper left quadrant of (**a**): here the MPs are grouped with different polygonal forms. MPs labelled as AI9 and AI10, reported in red colour, correspond to red-appearing areas in the funerary shroud. The black rectangle groups the MPs from the funerary shroud corresponding to the black inscriptions. The green pentagon groups the MPs on the brownish coloured inscriptions, while the red hexagon assembles the brownish coloured spots; the latter is probably connected to the “foxing” damage that is due to degradation processes. (**d**) Enlargement of (**a**) around the zero values for PC1 and PC2.

The algorithm used is the singular value decomposition (SVD), with the cross-validation method, and mean centre data as a model input to obtain a total of three components which contribute to the largest variance in elemental composition without reference to prior knowledge^27–29^. Furthermore, no weighting corrections (i.e. standard deviation) are applied to the variables; one runs the risk of inflating noise in wavelengths with little information. Indeed, spectral data are generally not weighted in order not to reduce the high variance variables^30^. Pre-processing analyses regarded only the baseline offset. Results are organised in a matrix of 82 × 15 entries, with respect to the inscriptions category (Fig. 4). Figure 4 shows the PCA; the two principal components (PCs) are extracted, representing 98% of the total variance (PC1: 93% and PC2: 5%). In the scores plot (Fig. 4a), six measured points (labelled as NI4, RI1, RI3, TI3, SI2, SI3) are separated by the rest of the MPs that show a more uniform composition, and this can be explained by a larger quantity of ink release during the hand-writing on these six points. The present analysis shows that the overall elemental composition of the MPs on the inscriptions is homogeneous. Here, the first principal component (PC1) is attributed to the release of ink from the nib (that increases from left to right). Figure 4b reports the PCs “loading plot” showing the correlation among the detected chemical elements. The main finding from the figure is that Fe is statistically correlated with Mn, since both are positively weighted in the first principal component. Calcium is in the lower right quadrant and MPs located in the same quadrant in Fig. 4a are those where Ca content is higher; they are located in the areas of the textiles that appears whiter, such as the MP labelled as SI3 (see the Table S1). In ancient Egypt, white painting pigments were used for their adhesiveness properties, such as CaMg_3_(CO_3_)_4_ (Huntite)_,_ CaCO_3_ (Calcite) and CaSO_4_·2H_2_O (Gypsum). The white appearance could reflect the presence of these compounds; for this reason, PC2 is attributed to the state of conservation or level of damage of the textiles.

As some MPs fall into close but distinct groups on the plot, Fig. 4c is a zoom of the overall loading plot of Fig. 4a in the region of the upper left quadrant. The MPs with the “A” as initial capital letter (black and red-appearing points, respectively), represent those MPs belonging to a single fragment from the funerary shroud that is from a different and earlier relic (cf. Introduction). The PCA analysis shows that the ink on the shroud presents low percentage of Fe that could be a contaminant. The MPs reported in red in Fig. 4c (labelled as AI9, AI10) refer to points, where a red pigment is observed, while the group enclosed by a green pentagon (labelled as BI) refers to MPs along a brownish inscription of a textile from the tomb of Kha (S.8532, see Table S1). Although the two groups belong to different relics and the pigments appear visually different, they present a comparable chemical composition, which indicates that both are based on ochre. Finally, the group enclosed within the red hexagon in Fig. 4c, is considered as a separate case. These MPs are characterised by brownish coloured spots (probably connected with the “foxing” damage, due to degradation processes) and are grouped together by the PCA. It can be noticed that PCA also shows the grouping of MPs from the same textile; this peculiarity can be attributed to the influence of the storage conditions (moist atmosphere). Figure 4d depicts an enlargement of the lower right part of Fig. 4a. It represents a separated grouping since here the MPs cannot be grouped as a function of the storage, as this is probably linked to the textile’s advanced state of degradation at these points. In this sense, negative PC2 values are attributed to increasing level of damage, which may arise from metal-catalysed oxidation by the excess of un-complexed iron(II), photo degradation of cellulose as the acid-catalysed hydrolysis, or brown degradation products of the ink itself.

## Discussion

The results demonstrate that the inks used for the inscriptions on the textiles of Kha are likely based on ochre, which consisted of three parts: the principle colour-producing component (hydrous or anhydrous iron oxide), the secondary or modifying colour component (manganese oxide) and the base carrier of the colour (earth). They appear similar to and share certain characteristics with iron gall inks, but cannot be classified as such, since the latter is based on iron/copper salts (green and blue vitriol) and not iron oxides. However, unlike iron gall ink that is an acidic solution, ochre is stable and does not cause “ink burns”, which suggest that tannins were added to the mixture as well. That not only iron salts were used in combination with tannins to produce a black/brown writing fluid, but also ochre, is suggested by anthropological studies of the black dyes employed by the Navajo Indians in the early twentieth century^31,32^. They made a black colour by boiling leaves and twigs of the aromatic sumac, the tannin yielding body, which was mixed with roasted yellow ochre and gum. Our results suggest that the ancient Egyptians used a similar mixture around 3400 years ago. Why this mixture was employed is not known, but perhaps it was more resistant than other ink solutions, e.g. carbon ink, to water and friction, since clothes intended for daily use were exposed to both on a regular basis. In order to answer this and other questions with any kind of certainty further experimental work, including extended molecular spectroscopy and isotopic analysis techniques to assess composition and provenance of the iron compounds, is needed, and the black inks on inscribed other types objects from the tomb of Kha, e.g. ceramics, papyri and wood, should be analysed^33^.

Full details of the Methods are reported in the Supplementary Information.

## Supplementary information

Supplementary Information
