# Author Correction: Egyptian metallic inks on textiles from the 15^th^ century BCE unravelled by non-invasive techniques and chemometric analysis

**DOI:** 10.1038/s41598-019-54735-5

**Published:** 2019-11-26

**Authors:** G. Festa, T. Christiansen, V. Turina, M. Borla, J. Kelleher, L. Arcidiacono, L. Cartechini, R. C. Ponterio, C. Scatigno, R. Senesi, C. Andreani

**Affiliations:** 1 CENTRO FERMI - Museo Storico della Fisica e Centro Studi e Ricerche “Enrico Fermi”, Piazza del Viminale 1, 00184 Rome, Italy; 2 CNR – Istituto per i Processi Chimico-Fisici (IPCF), Viale Ferdinando Stagno d’Alcontres 37, Messina, Italy; 3 CNR- Istituto di Scienze e Tecnologie Molecolari (ISTM), Via Elce di sotto 8, 06123 Perugia, Italy; 4 Museo Egizio di Torino, Via Accademia delle Scienze 6, 10123 Turin, Italy; 5 Supreme Council for Archeology of Piedmont, P.zza S. Giovanni 2, 10122 Torino, Italy; 6 STFC, Rutherford Appleton Laboratory - ISIS neutron and muon Facility, Didcot, OX11 0QX United Kingdom; 7 University College London, Institute of Archaeology, 31-34 Gordon Square, Kings Cross, London, WC1H 0PY United Kingdom; 8 Università degli Studi di Roma Tor Vergata, Dipartimento di Fisica and NAST Centre, Via della Ricerca Scientifica 1, 00133 Rome, Italy; 9 Università degli Studi di Roma Tor Vergata, Dipartimento di Scienze e Tecnologie Chimiche, Via della Ricerca Scientifica 1, 00133 Rome, Italy

Correction to: *Scientific Reports* 10.1038/s41598-019-43655-z, published online 13 May 2019

The Author Contributions section of this Article in incorrect.

“G.F. participated to the experimental campaigns, coordinated the data analysis through the integration of different techniques and contributed to the interpretation of the results. T.C. performed UV/NIRR imaging experiment and data analysis, contributed the theoretical discussion of the results and to the writing of the manuscript. V.T. participated to the experimental campaigns and was responsible of transport of the artefacts from Turin to Oxfordshire and their handling at the ISIS pulsed neutron source. M.B. participated to the experimental campaigns and contributed to the theoretical discussion. J.K. contributed to the interpretation of the neutron results and to the writing of the manuscript. L.A. participated to the PGAA measurements and performed the PGAA data analysis. L.C. performed XRF measurements and contributed to the discussion of XRF data analysis. R.C.P. performed the Raman experiment and relative data analysis and participated to the PGAA measurements. C.S. analysed the XRF data and performed the Principal Component Analysis and Box Plot Analysis. R.S. coordinated the PGAA measurements, contributed to the discussion of the PGAA results and to the writing of the manuscript. C.A. proposed and coordinated the experimental measurements and participated to the writing of the manuscript.”

should read:

“G.F. participated to the experimental campaigns, coordinated the data analysis through the integration of different techniques and contributed to the interpretation of the results. T.C. contributed the theoretical discussion of the results and to the writing of the manuscript. V.T. participated to the experimental campaigns and was responsible of transport of the artefacts from Turin to Oxfordshire and their handling at the ISIS pulsed neutron source. M.B. participated to the experimental campaigns and contributed to the theoretical discussion. J.K. contributed to the interpretation of the neutron results and to the writing of the manuscript. L.A. participated to the PGAA measurements and performed the PGAA data analysis. L.C. performed XRF measurements and contributed to the discussion of XRF data analysis. R.C.P. performed the Raman experiment and relative data analysis and participated to the PGAA measurements. C.S. analysed the XRF data and performed the Principal Component Analysis and Box Plot Analysis. R.S. coordinated the PGAA measurements, contributed to the discussion of the PGAA results and to the writing of the manuscript. C.A. proposed and coordinated the experimental measurements and participated to the writing of the manuscript.”

In addition, the Acknowledgements section of this Article is incomplete.

“This research is partially supported by CNR, within the CNR-STFC Agreement 2014–2020 (N. 3420), concerning collaboration in scientific research at the ISIS Spallation Neutron Source, and by the ARKHA (ARchaeology of the invisible: unveiling the grave-goods of KHA) project. Our thanks go out to Christian Greco and Stephen Hull for fruitful discussions and suggestions, to Tea Ghigo of the Bundesanstalt für Materialforschung und –prüfung (BAM) in Berlin for providing us with UV and NIR images of the textiles, and to the ISIS team of IMAT. Financial support by the Access to Research Infrastructures activity of E-RIHS.it (www.e-rihs.it) is also gratefully acknowledged.”

should read:

“This research is partially supported by CNR, within the CNR-STFC Agreement 2014–2020 (N. 3420), concerning collaboration in scientific research at the ISIS Spallation Neutron Source, and by the ARKHA (ARchaeology of the invisible: unveiling the grave-goods of KHA) project. Our thanks go out to Christian Greco and Stephen Hull for fruitful discussions and suggestions, and to the ISIS team of IMAT. We are very grateful to Tea Ghigo of the Bundesanstalt für Materialforschung und –prüfung (BAM) in Berlin who performed the UV/IRR experiments and data analysis and provided us with the images published in fig. 2. Financial support by the Access to Research Infrastructures activity of E-RIHS.it (www.e-rihs.it) is also gratefully acknowledged.”

