# Supplementary material for: Egyptian metallic inks on textiles from the 15th century BCE unravelled by non-invasive techniques and chemometric analysis
Source: Sci Rep. 2019 May 13;9:7310. doi: 10.1038/s41598-019-43655-z (PMC6513853; doi:10.1038/s41598-019-43655-z)
Supplement: Supplementary file 1 — Supplementary Information [file 41598_2019_43655_MOESM1_ESM.pdf]

Supplementary Information for

Egyptian metallic inks on textiles from the 15th century BCE unravelled by non-invasive techniques and chemometric analysis.

G. Festa[#], T. Christiansen[*], V. Turina[*], M. Borla[+], J. Kelleher[£], L. Arcidiacono[#], L. Cartechini[^], R.C. Ponterio[%], C. Scatigno[°$], R. Senesi[°#%]and C. Andreani[°#%]

[#]CENTRO FERMI - Museo Storico della Fisica e Centro Studi e Ricerche "Enrico Fermi", Piazza del Viminale 1, 00184, Rome, Italy

[%]CNR – Istituto per i Processi Chimico-Fisici (IPCF), Viale Ferdinando Stagno d'Alcontres37, Messina, Italy

[^]CNR- Istituto di Scienze e Tecnologie Molecolari (ISTM), Via Elce di sotto 8, 06123 Perugia, Italy

[*]Museo Egizio di Torino, Via Accademia delle Scienze 6, 10123, Turin, Italy

[+] Supreme Council for Archeology of Piedmont, P.zza S. Giovanni 2, 10122, Torino, Italy

[£]STFC, Rutherford Appleton Laboratory - ISIS neutron and muon Facility, Didcot, OX11 0QX, United Kingdom

[°]Università degli Studi di Roma Tor Vergata, Dipartimento di Fisica and NAST Centre, Via della Ricerca Scientifica 1, 00133, Rome, Italy

[$]Università degli Studi di Roma Tor Vergata, Dipartimento di Scienze e Tecnologie Chimiche, Via della Ricerca Scientifica 1, 00133, Rome, Italy

Email: *joe.kelleher@stfc.ac.uk*, *roberto.senesi@uniroma2.it*

This PDF file includes:

Supplementary text
Figs. S1 to S2
Tables S1 to S2
References for SI reference citations

Methods.

Samples. Table S1 shows a list of the 19 linen textiles that were investigated and reports visible light pictures and X-Ray fluorescence (XRF) measurement points. Tunics are labelled with blue, loincloth with red and inscriptions with green. Conservation issues affect some of the textiles, the main morphologies of degradation being organic and mechanical damage such as fibre breaks. Some of them have been restored with vinyl glue [1]. Chromatic alterations (coloured spots) also appear over macroscopic areas. A new identification acronym (ID) that identifies each XRF measurement point is also reported in the Table.

Item ID		MP	New ID	Item ID		MP	New ID
S.5065/2	AS	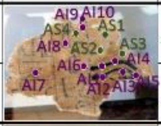	AS1, AS2,…AS4	S.8576	ML	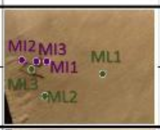	ML1, ML2, ML3
S.5065/2	AI		AI1, AI2,…AI10	S.8576	MI		MI1, MI2, MI3
S.8532	BT	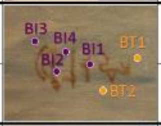	BT1, BT2	S.8578	NL	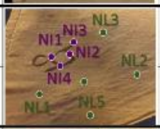	NL1, NL2, NL5
S.8532	BI		BI1, BI2,…BI4	S.8578	NI		NI1, NI2,…NI4
S.8534	CT	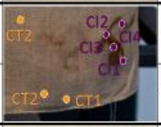	CT1, CT2, CT3	S.8587	OT	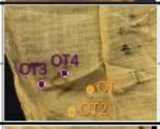	OT1, OT2
S.8534	CI		CI1, CI2,…CI4	S.8587	OI		OI1, OI2
S.8535	DT	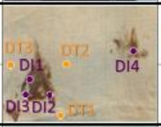	DT1, DT2, DT3	S.8613-15	PL	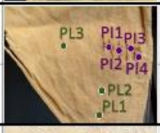	PL1, PL2, PL3
S.8535	DI		DI1, DI2,…DI4	S.8613-15	PI		PI1, PI2,…PI4
S.8536	ET	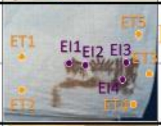	ET1,ET2,… ET5	S.8613-19	QL	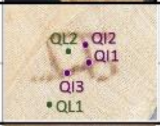	QL1, QL2
S.8536	EI		EI1, EI2,…EI4	S.8613-19	QI		QI1, QI2, QI3
S.8538	FT	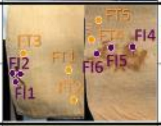	FT1, FT2,…FT7	S.8613-26	RL	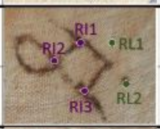	RL1, RL2
S.8538	FI		FI1, FI2,…FI9	S.8613-26	RI		RI1, RI2, RI3
S.8542	GT	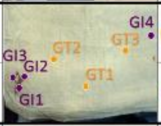	GT1, GT2, GT3	S.8617-6	SL	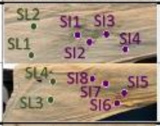	SL1, SL2,…SL4
S.8542	GI		GI1, GI2,…GI4	S.8617-6	SI		SI1, SI2,…SI8
S.8545	HT	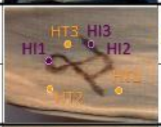	HT1, HT2, HT3	S.8617-10	TL	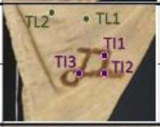	TL1, TL2
S.8545	HI		HI1, HI2, HI3	S.8617-10	TI		TI1, TI2, TI3
S.8546	IT	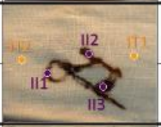	IT1, IT2	S.8617-11	UL	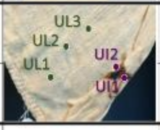	UL1, UL2, UL3
S.8546	II		II1, II2, II3	S.8617-11	UI		UI1, UI2, UI3
S.8565	LT	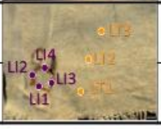	LT1, LT2, LT3	-	-	-	-
S.8565	LI		LI1, LI2,…LI4	-	-		-

Table S1. Description of the measurement points of the X-ray fluorescence (XRF) investigation on the Egyptian textiles. The Egyptian textiles were divided into the three main classes: tunics (T), loincloths (L), and inscriptions (I). The description of the assigned labels identifying the XRF measurement points (MPs) in the particular object is reported in the Table. The first column identifies the identification acronym (ID) that label the inventory number of each object in the catalogue of the Museo Egizio. The second column is the arbitrary assignment, progressive capital letter (A, B, …, U), associated with each textile typology (i.e. tunic [T], loincloth [L], inscription [I]). The last column describes the single MP as progressive number reported in the corresponding image of the investigated object; thus, for instance AS indicates the unpainted funerary shroud while AI indicates the inscriptions on the shroud.

Non-invasive analytical techniques. A synergic use of the four following techniques was applied to the study of the investigated Egyptian textiles.

Ultraviolet (UV) reflected imaging and infrared reflectography (IRR). A preliminary characterisation of the inks inscribed on four textiles (S.5065/2, S.8535, S.8536 and S.8578) was done using UV reflected imaging and IRR. For our investigations we used a Dino Lite microscope [2]. The microscope features built-in light emitting diode (LED) illumination at 395 nm and 940 nm and a customised external white-light source mounted on the microscope stand.

Portable X-Ray fluorescence (XRF). In order to characterize the elemental composition of the Egyptian textiles, 143 measurements were carried out on 19 ancient objects. A portable XRF analyser (Elio XGLab [3]) was used for these measurements; it was composed of a large area Silicon Drift Detector (25 mm^2) with resolution of 130 eV at MnKα with 10 kcps input photon rate (high-resolution mode), 170 eV at MnKα with 200 kcps input photon rate (fast mode). The acquisition live time for each measurement is 40 sec. It has a fast (USB 2.0) 8k channels MCA with high resolution and high count-rate capability. Its excitation source was a transmission X-Ray generator with a Rh anode operated at 100 μA and 40 kV; the beam was collimated to a spot diameter on the surface of about 1 mm^2. It has two pointing lasers (axial and focal), a microscope camera permitting field adjustment on the region of analysis. Spectra are reported in Figure S1.

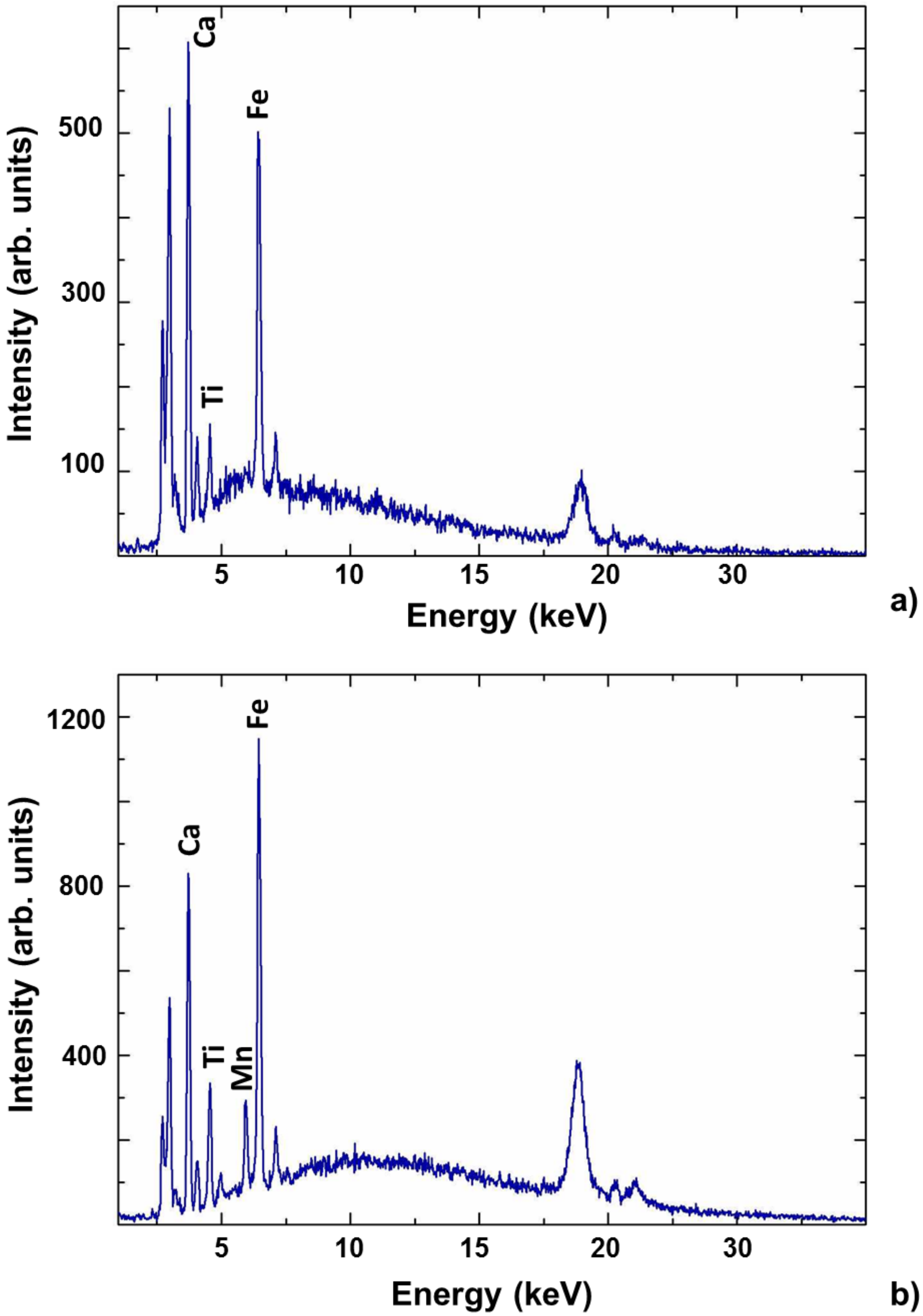

Fig. S1 - XRF spectrum of both a) textile and b) ink of the S.8535 object and the peak labelling [4].

Handled Raman spectroscopy. 120 spectra were collected by a BRUKER BRAVO Handheld Raman spectrometer using a Duo Laser excitation ranging from 700 to 1100 nm during the acquisition (BRAVO with Sequentially Shifted Excitation SSE™ – Bruker) in order to investigate the linen bulk, the coloured spot and the brownish areas due to degradation processes (i.e. textile S8617-6). DuoLaserTM excitation makes a spectral range of 300 – 3200 cm^{-1}. Figure S2 shows the spectra of both the textile and the ink complex for S8535 (representative example). The spectrum for the linen (Fig. S2b) textile shows Raman bands at 2093, 1375, 1326, 1278, 1123 and 1097 (strong), 535, 466, 435, and 384 (strong) cm^{-1}, characteristic peaks of cellulose [5]. The spectrum for the ink complex (Fig. S2a) shows Raman bands at 1515 (strong), 1482, 1208 (weak), 618 (weak) cm^{-1} (apart from the peaks that overlap with the cellulose spectrum). In the range of 600-800 cm^{-1} a signal enhancement is shown indicating the probable presence of iron oxides and oxyhydroxides [6–7].

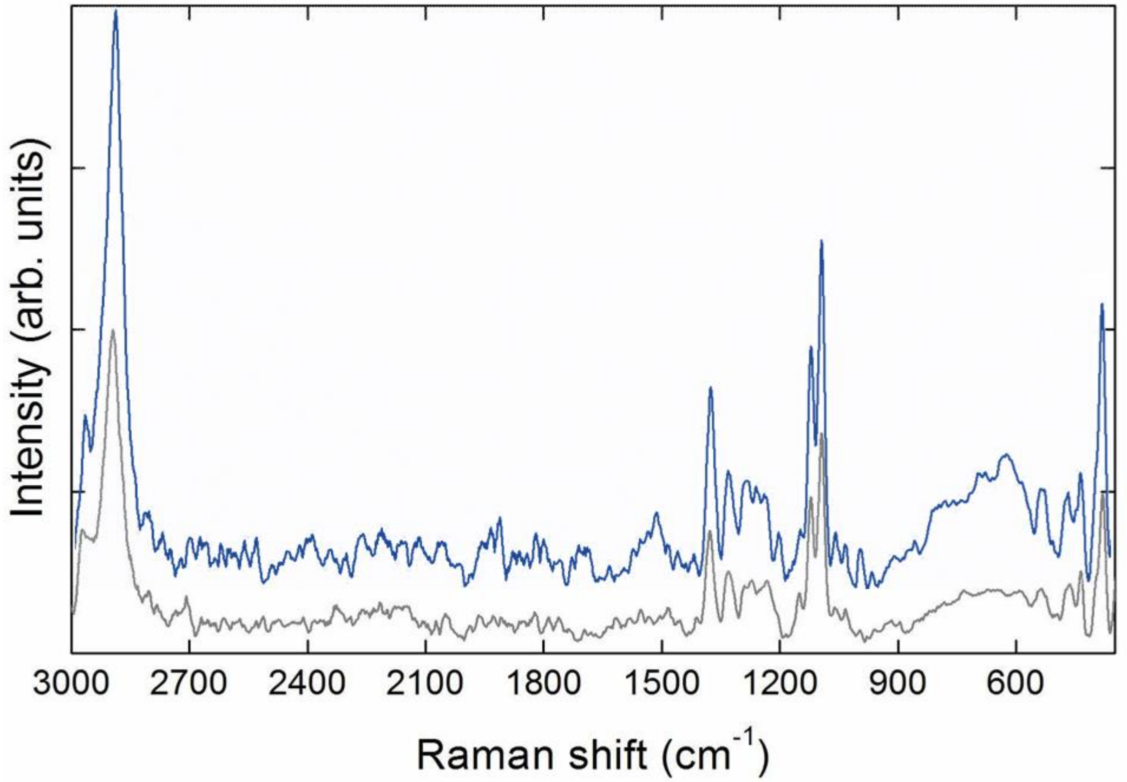

Fig. S2 - Raman spectra of both the ink (blue colour) and the textile (grey colour) of the S.8535 object.

Prompt gamma activation analysis (PGAA). Two linen textiles (S.8578 and S.8576) were analysed to detect the isotopic composition of the investigated objects. The incident neutron beam was of 10 cm x 10 cm in correspondence of the inscriptions and the measurements were the mean of the irradiated area (results of the measurements are reported in Table 2-SI). PGAA is a nuclear analytical technique based on radiative capture of neutrons, which uses the characteristic prompt γ-ray spectrum emitted during irradiation of the sample [8]. Prompt gamma energy is characteristic of the emitting nuclide, while their intensities are proportional to the number of atoms of a given nuclide present in the irradiated area. Generally, a nuclide emits a large number (several hundreds) of prompt gamma rays at different energies, and the spectrum is usually complicated [9-13]. In the present work, γ-ray spectra were recorded using an ORTEC - GMX15 HPGe detector [14] with efficiency varying between 25% at 60 keV and 3-7% at 1332 keV. The HPGe detector was located at 1.5 m from the sample position; its entrance window was a 0.3 μm-

thick, ion-implanted contact, extending the lower range of energies to about 3 keV. The electronic chain of the data acquisition used the ORTEC-DSPEC50 [14] as high voltage supply and multi-channel analyser. The analysis was performed using ORIGIN-Lab [15]; isotopes' identification was carried out through the comparison of two γ activation databases, i.e. Molnar [8] and the International Atomic Energy Agency [16], both including prompt and delayed γ-rays. A semi-quantitative analysis was performed normalizing the peak area by the γ cross section and the (n,γ) cross-sections weighed by the absolute flux measured on IMAT beamline, where the measurements were carried out. The relative percentages reported in Table S2 were obtained for each element's γ line with the higher γ cross-section and through the normalization respect to the most intense. Results of PGAA are shown in Table S2.

**ID**	**^{24}Mg**	**^{31}P**	**^{35}Cl**	**^{40}K**	**^{56}Fe**	**^{63}Cu**	**^{65}Cu**	**^{75}As**
**S.8576**	0.12	0.44	100	68.6	6.56	0.22	5.15	10.8
**S.8578**	100	6.09	-	-	23.7	0.089	2.18	1.026

Table S2. Prompt Gamma Activation Analysis results. The relative percentages of detected elements are obtained through the normalization of peaks with respect to the most intense. The errors on measurements are 3%.

Statistical approach.
Statistical methods such as distribution analysis with box plot and chemometric tools – principal component analysis (PCA) – were used in the present work [17, 18]. The boxes were created, as the first step, in order to identify the general trend and statistical distribution of each chemical element, as well as the cross correlation between the location of the measurement points and the detected chemical elements. The box chart distribution analysis was performed following the "Box and Whiskers" representation, where the chemical elements were plotted on the x-axis and the raw measured distribution was reported in the y-axis [19]. In the present study, the "Box and Whiskers" representation was used for the identification of the "key chemical elements" for each category (tunics, loincloths and inscriptions). Conventionally, the box shows the median, the distribution spread, skew and percentile values 5% and 93% (also the probably outliers) by whiskers representation [19]. The chemometric analysis refers to the PCA, a data multivariate analysis used for the evaluation of the variables' correlation and their relevance, reducing the dimensionality of the data. In our specific case, the PCs were performed using the data matrix (143x15) from the analysis of the XRF spectra and three sub-matrices (41x15, 20x15, 82x15) were generated by dividing results for tunics, loincloths and inscriptions. A singular value decomposition (SVD) algorithm [19]) and the cross-validation method were applied obtaining three main components. The present work was performed through Unscramble X version 9.5 (chemometric software package - Camo, Woodbridge, NJ, USA) [20].

References

1. Unpublished report by Radelet, P. (2015)
2. http://www.dino-lite.com/products_detail.php?index_m1_id=9&index_m2_id=35&index_id=127
3. https://www.xglab.it/compact-portable-xrf-spectrometer-elio.shtml
4. García-Florentino, C. *et al*. A fast in situ non-invasive approach to classify mortars from a construction of high historical value. *Microchemical Journal* **133**, 104–113, doi: 10.1016/j.microc.2017.03(2017).

5. Szymańska-Chargot, M., Cybulska, J. & Zdunek, A. Sensing the structural differences in cellulose from apple and bacterial cell wall materials by Raman and FT-IR spectroscopy. *Sensors* **11**(6), 5543–5560, doi:10.3390/s110605543 (2011).
6. Lee A.S., Mahon, P.J. & Creagh, D.C. Raman analysis of iron gall inks on parchment. *Vibrational Spectroscopy* **41**(2), 170–175, doi:10.1016/j.vibspec.2005.11.006 (2006).
7. de Faria D.L.A., Venâncio Silva S & de Oliveira M.T. Raman microspectroscopy of some iron oxides and oxyhydroxides. *Journal of Raman Spectroscopy* **28**(11), 873-878, doi:10.1002/(SICI)1097-4555(199711)28:11<873::AID-JRS177>3.0.CO;2-B (1997).
8. Molnar G.L. *Handbook of Prompt Gamma Activation Analysis with Neutron Beams* (Kluwer Academic Publishers 2004).
9. Pietropaolo, A. *et al.* Single-crystal diamond detector for time-resolved measurements of a pulsed fast-neutron beam, *Europhysics Letters* **92**(6), 68003 (2011).
10. Andreani C. *et al.* Electron-volt spectroscopy at a pulsed neutron source using a resonance detector technique. *Nuclear Instruments and Methods in Physics Research Section A: Accelerators* **481**(1–3), 509–520 (2002).
11. Tardocchi, M *et al.* Cadmium-Zinc-Telluride photon detector for epithermal neutron spectroscopy-pulse height response characterisation, *Nuclear Instruments and Methods in Physics Research Section A: Accelerators* **526**(3), 477–492 (2004).
12. Schooneveld, E.M., Mayers, J., Rhodes N.J., Pietropaolo, A. & Andreani, C. Foil cycling technique for the VESUVIO spectrometer operating in the resonance detector configuration. *Review of Scientific Instruments* **77**(9), 095103 (2006)
13. Belgya, T., Kis, Z., Szentmiklósi, L., Kasztovszky, Z., Festa, G. & Andreanelli, L. A new PGAI-NT setup at the NIPS facility of the Budapest Research Reactor, *Journal of Radioanalytical and Nuclear Chemistry* **278**(3), 713–718 (2008).
14. http://www.ortec-online.com/
15. http://www.originlab.com/
16. IAEA, International Atomic Energy Agency, Database for Prompt Gamma-ray Neutron Activation Analysis, https://nucleus.iaea.org/Pages/pgaa-iaea.aspx, 2013
17. McGill, R., Tukey J.W. & Larsen, W.A. Variations of box plots. *The American Statistician* **32**, 12–16 (1978).
18. Jolliffe, I.T. *Principal Component Analysis* [2nd]: 487, doi:10.1007/b98835 (Springer-Verlag 2002).
19. François, H., Lê, S. & Jérôme, P. *Exploratory Multivariate Analysis by Example Using R*. (CRC press 2009)
20. http://www.camo.com/rt/Products/Unscrambler/unscrambler.html
